# Female homicides in Brazil: global burden of disease study, 2000–2018

**DOI:** 10.1016/j.lana.2024.100935

**Published:** 2024-11-07

**Authors:** Nadia Machado de Vasconcelos, Juliana Bottoni de Souza, Adauto Martins Soares Filho, Polyanna Helena Coelho, Sofia Reinach, Caroline Stein, Crizian Saar Gomes, Luisa Sorio Flor, Emmanuela Gakidou, Antonio Luiz Pinho Ribeiro, Deborah Carvalho Malta

**Affiliations:** a Federal University of Minas Gerais, Belo Horizonte, Minas Gerais, Brazil; b Brazilian Ministry of Health, Brasilia, Federal District, Brazil; c Civil Police of Minas Gerais, Belo Horizonte, Minas Gerais, Brazil; d Vital Strategies, São Paulo, São Paulo, Brazil; e University of Washington, Seattle, WA, USA

**Keywords:** Violence against women, Gender-based violence, Brazil, Homicide, Public health surveillance

## Abstract

**Background:**

Female homicides are a public health-relevant issue, and its spatial distribution may evidence socioeconomic vulnerabilities. This study aims to analyze the temporal and spatial trends of female homicides in Brazil and investigate socioeconomic-demographic factors associated with it.

**Methods:**

This is an ecological, descriptive, and analytical epidemiological study investigating the age-standardized female homicide rate in all Brazilian municipalities between 2000 and 2018, divided into three periods. Spatial and temporal analyses were conducted using the Global Moran's Index and LISA to identify clusters of high and low rates. Rates were also calculated by population size and means of violence across macro-regions. For the last period, a multivariable linear regression model analyzed the association of female homicide rates with social, economic, and geographic factors.

**Findings:**

Female homicide rates in Brazil remained high during the studied period, with differences in trends between regions. Among the potentially associated factors, it was observed that male homicide rate, the high percentage of violent deaths among black women and those with low levels of education, in addition to the low Gross Domestic Product (GDP) *per capita*, were positively associated with female homicide, whereas larger cities were negatively associated.

**Interpretation:**

These findings show that Brazil is a country with a high risk of female homicide. Nevertheless, the vulnerability of women is unequally distributed in the country. Female homicides are mostly caused by domestic conflicts but can also be influenced by changes in the urban and social contexts.

**Funding:**

This project is funded by the 10.13039/100000865Bill & Melinda Gates Foundation.

**Research in context**

**Evidence before this study**

A search was conducted in PubMed, Embase, and Scopus using the following terms (“Domestic Violence” OR “Battered Women” OR “Abused Women” OR “Domestic and Sexual Violence Against Women” OR “Offenses Against Women” OR “Crimes Against Women” OR “Violence Against Women” OR “Gender-Based Violence”) AND (“Homicide” OR “Fatal violence” OR “Violence death” OR “Femicide” OR “Feminicide” OR “Mortality”) AND (“Brazil” OR “Brasil”). Studies from 2000 onwards with free full text were selected, with no language restriction. The final selection included 27 studies, of which 02 focused on homicides in pregnant women and another 13 addressed the issue regionally, without representing the Brazilian population. Of the remaining 11 studies, all used the Brazilian Mortality Information System (SIM, in Portuguese) as their database, most conducted analyses at the state level and on women of reproductive age (10–49 years old), one examined mortality from injuries, and another looked at rates only for homicides by firearms. Additionally, 03 studies analysed the relationship between violent deaths and previous violence notifications. These studies indicated a high rate of female homicide in Brazil, and that women with a history of violence notification are at greater risk of death by any cause, but mainly from assault.

**Added value of this study**

This is the first study to conduct a temporal analysis of female homicide rates in Brazil at the municipal level, evaluating almost two decades, using official data from the country, and applying the Global Burden of Disease (GBD) methodology. Additionally, associations of deaths with social, demographic, and economic factors were analysed. The study findings show that Brazil experienced a decline in female homicide rates during the study period, mainly attributable to a decrease in deaths by firearms. However, the study also revealed that the decline occurred unevenly across the territory, primarily concentrated in larger municipalities of the Southeast macro-region. Lastly, the study demonstrated an association between female homicide and male homicide, black race, lower educational level, and lower GDP *per capita*.

**Implications of all the available evidence**

Despite the decline in female homicide rates in recent decades, Brazil still ranks among the most violent countries for women worldwide. Within Brazil, the decrease in this rate occurs unevenly, in terms of spatiality and sociodemographic factors. The data from this study provides evidence that can inform public policies aimed at preventing violence against women. Regional differences highlight the need to consider regional disparities and inequalities in the development, implementation, and monitoring of public policies. The association of homicide with sociodemographic factors reinforces the need for intersectoral measures to address violence, with improvements in income and education accessibility for women and an intersectional perspective of race and gender.

## Introduction

Interpersonal violence, defined as “*the intentional use of physical force or power against other persons by an individual or small group of individuals*”, is a serious cause of morbimortality worldwide and, in its most lethal form, it can manifest as intentional homicide.^1^ Although men are the majority affected by this type of crime, female homicide deserves special attention, as it carries specific socio-historical characteristics.^2^

Previous studies have shown that female homicides are mostly femicides, defined as the intentional killing of women with gender-related motivations. Femicides can take place in different situations.^3^ Global data show that female homicide occurs predominantly within the home environment, with more than half of these perpetrated by an intimate partner or family member.^4^^,^^5^ However, femicide also takes place in public spheres, where gender-motivated homicide can be related to armed conflicts, human and drug trafficking, and other forms of organized crime.^3^

Female homicide is influenced by social determinants, which, in turn, are directly associated with the spatial distribution of inequalities. Places with greater social and economic vulnerability, represented by the concentration of poverty, low levels of schooling, and lack of attention from the State, are at greater risk of homicides.^6^ Therefore, the spatial approach to violence becomes a valuable tool for evaluating the impact of social processes on the distribution of homicides and the particularities of female homicides.^7^ The possibility of unearthing patterns in critical areas can provide fundamental supporting evidence in violence prevention policies.

Eliminating gender-based violence is a key commitment of the 2030 Agenda for Sustainable Development from the United Nations. For Brazil, specifically, the goal is that, by 2030, there will be a one-third reduction in femicide rates.^8^ Understanding female homicide in terms of frequency and distribution is the first step towards defining its magnitude in the country and producing high-quality evidence to support public policies to address this type of violence effectively and specifically. Therefore, the present study aims to analyse the temporal and spatial trends of female homicide in Brazilian territory and investigate the possible social, economic, and demographic factors associated with it.

## Methods

### Design and data source

This is an ecological, descriptive, and analytical epidemiological study that investigated the age-standardized homicide rate among women in all Brazilian municipalities between 2000 and 2018.

The data for this study were extracted from the Brazilian Mortality Information System (SIM, in Portuguese) at the municipality level. The Global Burden of Disease (GBD) Study methodology was applied to the extracted data, namely: the distribution of causes of death from the original coding onto the GBD Cause of Death (CoD) list, disaggregation by sex and age, and redistribution of *garbage* codes.

#### Brazilian Mortality Information System (SIM)

The official mortality data in Brazil is processed and stored by the Ministry of Health, via the Mortality Information System (SIM), which is publicly available on the DataSUS website.^9^ Every death in the national territory should be recorded in this system, accompanied by pertinent information for conducting analyses on the nation's health status. The Ministry of Health has been engaged in systematic efforts to preserve the quality of this system, aiming to reduce underreporting and enhance the accuracy in classifying basic causes of death. Among the latter are the ill-defined and nonspecific causes, which are disease categories acknowledged in the International Classification of Diseases 10th Revision (ICD-10) but that should not be listed as primary causes of death. Despite the enhancements in the quality of SIM data over the last decade, in the three-year period from 2015 to 2017 there were almost 40% of deaths attributed to these so-called “*garbage* codes” in SIM,^10^ and in 2020 this percentage remained above 15%.^11^ Therefore, using SIM raw data for epidemiological analyses is not recommended, as the significant proportion of *garbage* codes undermines the accuracy of the mortality profile of the population.^10^

#### GBD methodology

The GBD Study applies a multi-step method to process mortality data, which has been fully detailed in previous studies.^12^^,^^13^ A summary overview of this methodology will be described for contextualisation. First, all causes of death are mapped from their original coding onto the GBD Cause of Death (CoD) list. Second, the data is split into detailed age and sex groups. After that, the causes of death are evaluated and those considered inconsistent (for example, ovarian cancer in men) are reassigned to the most probable underlying causes of death.^13^ The next step, which is the most impactful, is the redistribution of *garbage* codes. Briefly, for each group of diagnostically related *garbage* code, a specific array of potential underlying causes of death is identified, along with the proportion of deaths erroneously coded, which are then reallocated to each cause.^12^ Finally, noisy data due to stochastic variation are smoothed using a Bayesian noise-reduction algorithm^13^ and an underreporting correction factor is applied. All these steps are performed considering age, sex, location, and year.

### Variables

#### Outcome variable

The GBD organises the causes of death into progressively disaggregated levels. For the matter of this study, at the least disaggregated level (Level 1), there are the “Injuries”, which sub-divides into “transport injuries”, “unintentional injuries”, and “self-harm and interpersonal violence” (Level 2). In this last group, the causes are disaggregated into “self-harm”, “interpersonal violence”, “conflict and terrorism” and “executions and police conflict” (Level 3).

In the present study, to identify the outcome variable “female homicide”, the cause of death “interpersonal violence” was selected among women. To assess the means of violence, three categories were selected from the most disaggregated level (Level 4): violence by firearm, violence by sharp object, and violence by other means. “Sexual violence” is also a category at Level 4 of “interpersonal violence”. However, in the GBD methodology, this subtype of violence is considered only a cause of morbidity and, therefore, sexual violence that did not result in a fatal outcome is not included in the analysis.

#### Explanatory variables

Explicative variables were selected to verify possible associations with female homicides in the last studied triennium (2016–2018), as follows:

a) Male homicides *per* municipality: The male age-standardized homicide rate in the triennium (2016–2018), using the same SIM database adjusted by the GBD methodology, as used for the primary outcome.
b) Gross Domestic Product (GDP) *per capita* of the municipalities: The GDP *per capita* is calculated by dividing the GDP by the number of inhabitants of a given geographic space (in this study, municipalities) and measures how much of the GDP would be assigned to each individual of a municipality if all received equal quotas. The municipality-level GDP values are also publicly available and were extracted from the Brazilian Institute of Geography and Statistics (IBGE, in Portuguese) database. The average values of the last studied triennium were used for this study (2016–2018).
c) Level of education: The percentage of all female homicides involving women who had a lower education level (eight years of education or less) was used, according to data from SIM. The average values of the triennium studied (2016–2018) were used.
d) Race: The percentage of all female homicides in which the victims were black (here considered the combined number of black or brown women) was used, according to data from SIM. The average values of the triennium studied (2016–2018) were used. The data relating to race were collected from the SIM, and the guideline states that the colour of the deceased should be inquired of the family member or the individual responsible for providing the information. Five standardised options for race/skin colour are recognised in Brazil: white, black, brown, yellow, and indigenous. The yellow and indigenous are included in the total number of homicides; however, separate analyses for them were not conducted due to their low frequency (representing together less than 1% of all female homicides during the period).
e) Population size of the municipalities: This information is estimated and published by IBGE. The estimation for 2017 was used and categorized by whether they have more than 50,000 inhabitants (1) or fewer (0).
f) Women's protective services: Data from the Basic Municipal Information Survey (MUNIC, in Portuguese), 2018, conducted by the IBGE, considering the presence (1) or absence (0) of an executive body for women's policy in each municipality, namely: Office for Violence Against Women (VAW), council for women's rights, Specialized services for cop VAW and police station for women.

### Statistical analysis

The female age-standardized homicide rate, expressed per 100,000 women, was calculated for all the 5564 Brazilian municipalities, from 2000 to 2018, divided into three-year consecutive periods: 2000-2001-2002 (T1), 2009-2010-2011 (T2) and 2016-2017-2018 (T3).

The numerator included the average number of female homicides, which refers to deaths recorded for the “female” sex, across all ages (including children). The denominator included the average female population for each triennium, extracted from the database of population estimates from the Brazilian Ministry of Health.

To mitigate the impact of the age distribution within the municipal population, the standardised rates for age were calculated using the standard population from the GBD study as a reference (https://ghdx.healthdata.org/record/ihme-data/gbd-2019-population-estimates-1950-2019).

The three-year periods were used to minimise the fluctuations caused by using data from small areas. This approach was necessary because many municipalities (over 60%) have a population of less than 20,000 inhabitants and the small numbers could generate high variability in the rates.

An initial exploratory analysis was conducted to investigate the space-time trends of female homicide rates. For this, choropleth maps (maps that use colour to represent the data variation in each region) and spatial analysis of the municipalities were used. First, a spatial analysis was carried out by applying the Global Moran's Index, capable of assessing the spatial interdependence among all polygons that constitute the geographical expanse of Brazil and representing it with a singular value for the entire country. Next, the Local Indicator of Spatial Autocorrelation (LISA) was used to detect clusters among the Brazilian municipalities.^14^ To better visualize the results, only the High–High and Low–Low clusters were presented. The High–High clusters represent groups of neighbouring municipalities with a high female homicide rate, which means that each municipality in this group has a high rate of female homicide and is surrounded by other municipalities with high female homicide rates. By contrast, the Low–Low clusters are composed of groups of neighbouring municipalities that present low rates of female homicide.

For the descriptive analysis, female homicide rates and the 95% confidence intervals (95% CI) were calculated for each triennium, by population size and by means of violence, per macro-region of Brazil.

For the last triennium (2016–2018), a multivariable linear regression model was used to identify the possible association between the above-listed explanatory variables and female homicides. A multivariate linear regression was performed. All explanatory variables were included in the first model, and a Pearson correlation analysis was conducted, considering those with a p-value <0.05 as significant. Subsequently, a new analysis was performed with the variables that remained significant in Model 1, using the standard entry method. The final model was considered significant at the 5% level.

Data presentation and analysis were carried out using the statistical software RStudio, version 4.3.2.

### Ethical approvals

This study used secondary data from public databases and, therefore, did not require approval from the Research Ethics Committee, according to that outlined in Resolution no. 466/2012 from the Brazilian National Health Council.

### Role of the funding source

This project is funded by the Bill & Melinda Gates Foundation. The Foundation had no participation in the study design, in the collection, analysis, and interpretation of data, in the writing of the manuscript, nor in the decision to submit the paper for publication.

## Results

**Fig. 1:**
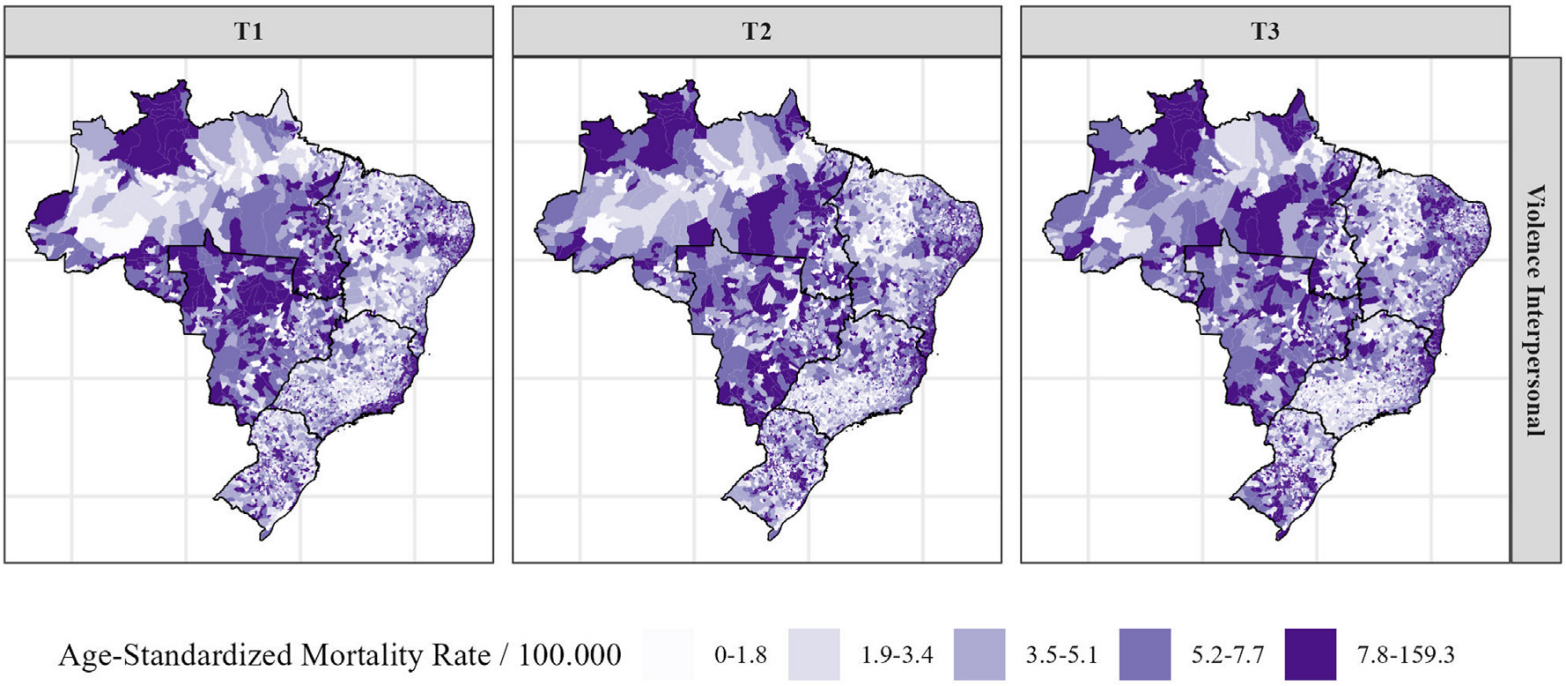
Age-standardized female homicide rates (per 100,000 women) in Brazilian municipalities, during the trienniums of 2000-2001-2002, 2009-2010-2011, and 2016-2017-2018.

Female homicide rates in Brazilian municipalities varied from zero to 159.3 per 100,000 women in the trienniums studied. Municipalities from the North and Midwest macro-regions of the country showed the highest rates throughout the entire studied period while the lowest rates were observed in the Southeast macro-region (Fig. 1).

**Fig. 2:**
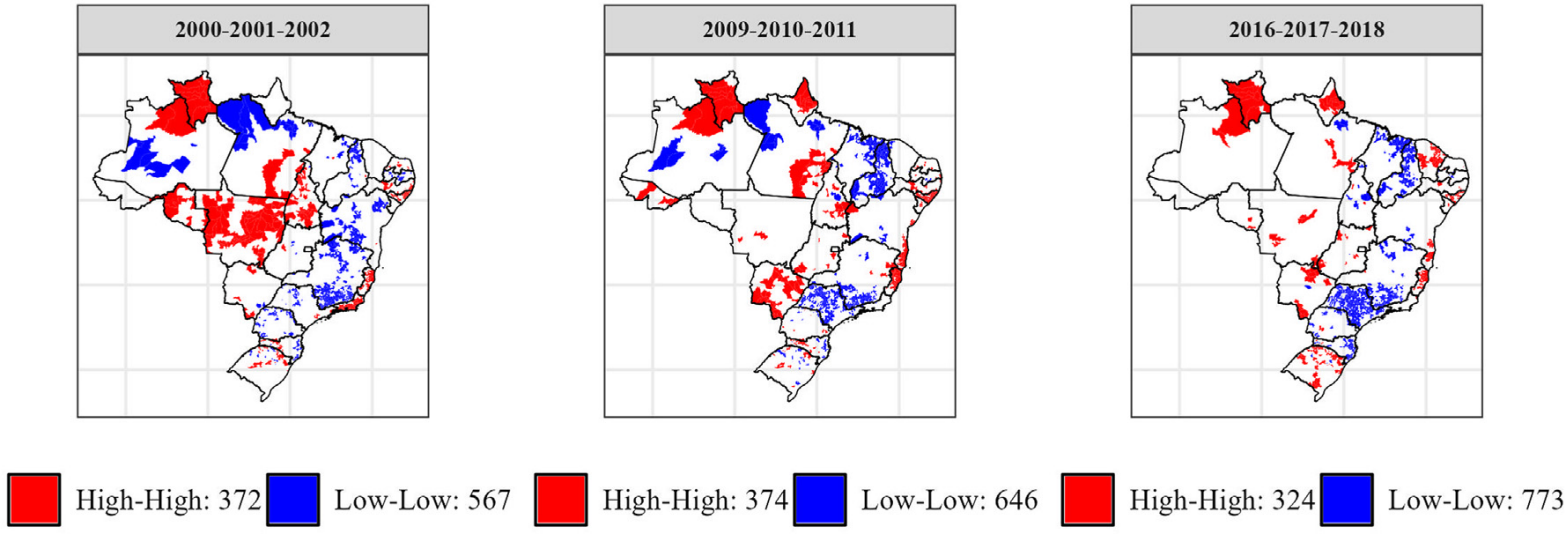
Space-time trends of female homicide rates in Brazilian municipalities, during the trienniums of 2000-2001-2002, 2009-2010-2011, and 2016-2017-2018.

Throughout the three trienniums, the municipalities located in the states of Roraima and Alagoas, the northeastern region of the state of Amazonas, the extreme southern region of Mato Grosso do Sul and the northern region of Espírito Santo were the ones that maintained “high–high” clusters. By contrast, only the municipalities in the northern region of Piaui and a small part of the southern region of Minas Gerais showed “low–low” clusters in all the trienniums (Fig. 2).

Throughout the studied period, Brazil went from a female age-standardized homicide rate per 100,000 women of 6.2 (95% CI: 6.1–6.4) in the first triennium to 5.4 (95% CI: 5.2–5.5) in the last one, with a relative reduction of 14.3% from 2000 to 2018 (Table 1). Considering the macro-regions of the country, female homicide rate decreased in the Southeast, with a percentage of relative change of −42.0% between T1 (7.0; 95% CI: 6.7–7.2) and T3 (4.0; 95% CI: 3.9–4.2), while it increased in the Northeast, with a percentage of relative change of 14.0% between T1 and T3, going from 5.9 (95% CI: 5.6–6.2) to 6.7 (95% CI: 6.4–7.0). The change in rates seems to be directly related to firearm homicides (Table 1). In Brazil, female homicide by firearms rate reduced from 3.4 (95% CI: 3.2–3.5) in the first triennium to 2.7 (95% CI: 2.6–2.8) in the last one, a relative reduction of 20.4%. In the Southeast macro-region, the female homicide by firearm rate had a relative reduction of 58.3%, going from 4.2 (95% CI: 4.0–4.4) in the first triennium to 1.8 (95% CI: 1.6–1.9) in the last one. Conversely, the female homicide by firearms rate in the Northeast macro-region went from 2.8 (95% CI: 2.6–3.0) to 3.8 (95% CI: 3.6–4.0), with a relative increase of 33.9% (Table 1).

**Table 1:** Age-standardized female homicide rates (per 100,000 women) with 95% confidence intervals and percentages of relative change (%) between trienniums T1, T2, and T3, stratified by means of violence and region.

Cause	Region	T1	T2	T3	T1–T2	T2–T3	T1–T3
Homicide by any means	Brazil	6.2 (6.1–6.4)	5.5 (5.4–5.7)	5.4 (5.2–5.5)	−11.5	−3.2	−14.3
	Midwest	6.4 (5.8–7.0)	6.4 (5.8–7.0)	6.1 (5.6–6.6)	−0.1	−4.3	−4.4
	Northeast	5.9 (5.6–6.2)	6.6 (6.3–6.9)	6.7 (6.4–7.0)	12.3	1.5	14.0
	North	5.9 (5.3–6.5)	6.0 (5.5–6.6)	6.6 (6.1–7.2)	1.7	10.5	12.4
	Southeast	7.0 (6.7–7.2)	4.6 (4.4–4.8)	4.0 (3.9–4.2)	−33.4	−12.9	−42.0
	South	4.7 (4.3–5.0)	5.2 (4.8–5.6)	5.2 (4.8–5.6)	11.3	0.4	11.7
Homicide by firearm	Brazil	3.4 (3.2–3.5)	2.7 (2.6–2.8)	2.7 (2.6–2.8)	−20.9	0.5	−20.4
	Midwest	3.0 (2.6–3.4)	2.8 (2.4–3.2)	2.8 (2.4–3.2)	−7.4	0.6	−6.9
	Northeast	2.8 (2.6–3.0)	3.4 (3.2–3.6)	3.8 (3.6–4.0)	20.9	10.7	33.9
	North	2.4 (2.0–2.8)	2.5 (2.2–2.9)	3.1 (2.8–3.5)	6.5	23.8	31.9
	Southeast	4.2 (4.0–4.4)	2.1 (2.0–2.3)	1.8 (1.6–1.9)	−49.0	−18.2	−58.3
	South	2.3 (2.0–2.6)	2.6 (2.4–2.9)	2.7 (2.4–3.0)	13.8	2.9	17.1
Homicide by sharp object	Brazil	1.3 (1.2–1.4)	1.4 (1.3–1.5)	1.3 (1.2–1.4)	4.4	−6.3	−2.3
	Midwest	1.9 (1.5–2.2)	2.0 (1.7–2.3)	1.8 (1.5–2.1)	7.3	−9.5	−2.9
	Northeast	1.6 (1.4–1.7)	1.6 (1.5–1.8)	1.5 (1.4–1.6)	3.6	−8.0	−4.7
	North	2.2 (1.8–2.6)	2.2 (1.8–2.5)	2.1 (1.8–2.4)	−0.3	−1.1	−1.4
	Southeast	1.0 (0.9–1.1)	1.0 (0.9–1.1)	0.9 (0.8–1.0)	0.2	−9.7	−9.5
	South	1.2 (1.0–1.4)	1.3 (1.1–1.5)	1.3 (1.1–1.4)	7.8	−1.6	6.1
Homicide by other means	Brazil	1.5 (1.4–1.6)	1.5 (1.4–1.6)	1.4 (1.3–1.5)	−4.9	−7.0	−11.5
	Midwest	1.5 (1.2–1.9)	1.6 (1.3–1.9)	1.5 (1.2–1.8)	4.9	−6.7	−2.2
	Northeast	1.5 (1.3–1.6)	1.6 (1.4–1.7)	1.4 (1.3–1.6)	5.5	−9.3	−4.4
	North	1.4 (1.1–1.7)	1.3 (1.1–1.6)	1.4 (1.1–1.6)	−3.4	3.3	−0.2
	Southeast	1.7 (1.6–1.9)	1.5 (1.4–1.6)	1.4 (1.3–1.5)	−15.1	−7.5	−21.4
	South	1.2 (1.0–1.4)	1.3 (1.1–1.5)	1.2 (1.1–1.4)	10.3	−2.6	7.5

Regarding the size of the cities within each macro-region, only Southeast municipalities showed significant changes in female homicide rates throughout the period analysed in this study. All municipalities with 20,000 or more inhabitants of this macro-region showed a decrease in the female homicide rates, which was even more pronounced for municipalities with more than 100,000 inhabitants, where a relative reduction of 50.6% between T1 and T3 was observed (from 8.1 (95% CI: 7.7–8.4) to 4.0 (95% CI: 3.8–4.3)) (Table 2).

**Table 2:** Age-standardized female homicide rates (per 100,000 women) with 95% confidence intervals and percentages of relative change (%) between trienniums T1, T2, and T3 stratified by region and population size.

Region	Population size	T1	T2	T3	T1–T2	T2–T3	T1–T3
Midwest	Up to 20,000	7.8 (6.6–9.0)	7.6 (6.4–8.7)	7.6 (6.5–8.7)	−2.6	0.0	−2.6
	More than 20,000–50,000	6.9 (5.1–8.8)	6.9 (5.3–8.5)	7.3 (5.7–8.9)	0.0	5.8	5.8
	More than 50,000–100,000	7.5 (4.8–10.2)	8.0 (5.9–10.1)	7.0 (5.0–9.1)	6.7	−12.5	−6.7
	More than 100,000	5.2 (4.3–6.0)	5.2 (4.5–6.0)	5.0 (4.3–5.6)	0.0	−3.8	−3.8
Northeast	Up to 20,000	5.7 (5.2–6.2)	6.1 (5.6–6.6)	6.2 (5.8–6.7)	7.0	1.6	8.8
	More than 20,000–50,000	5.5 (4.9–6.2)	6.2 (5.5–6.9)	6.7 (6.1–7.4)	12.7	8.1	21.8
	More than 50,000–100,000	5.9 (4.7–7.0)	7.3 (6.2–8.3)	7.0 (6.0–8.0)	23.7	−4.1	18.6
	More than 100,000	6.4 (5.8–6.9)	7.3 (6.7–7.8)	7.2 (6.7–7.7)	14.1	−1.4	12.5
North	Up to 20,000	7.3 (6.0–8.5)	6.1 (5.1–7.1)	6.4 (5.4–7.4)	−16.4	4.9	−12.3
	More than 20,000–50,000	5.2 (3.9–6.5)	6.1 (4.9–7.3)	6.3 (5.2–7.5)	17.3	3.3	21.2
	More than 50,000–100,000	5.6 (3.3–7.9)	6.3 (4.5–8.1)	7.0 (5.4–8.7)	12.5	11.1	25.0
	More than 100,000	5.4 (4.4–6.4)	5.8 (5.0–6.6)	6.9 (6.0–7.7)	7.4	19.0	27.8
Southeast	Up to 20,000	4.8 (4.3–5.3)	4.5 (4.0–4.9)	4.2 (3.7–4.7)	−6.3	−6.7	−12.5
	More than 20,000–50,000	5.7 (5.0–6.3)	4.2 (3.6–4.8)	3.7 (3.2–4.2)	−26.3	−11.9	−35.1
	More than 50,000–100,000	6.8 (6.0–7.5)	4.7 (4.1–5.3)	4.2 (3.6–4.7)	−30.9	−10.6	−38.2
	More than 100,000	8.1 (7.7–8.4)	4.8 (4.5–5.1)	4.0 (3.8–4.3)	−40.7	−16.7	−50.6
South	Up to 20,000	6.1 (5.3–6.8)	5.9 (5.2–6.6)	6.5 (5.8–7.3)	−3.3	10.2	6.6
	More than 20,000–50,000	3.9 (3.1–4.7)	4.8 (3.9–5.6)	4.6 (3.8–5.5)	23.1	−4.2	17.9
	More than 50,000–100,000	3.9 (2.9–4.9)	4.5 (3.6–5.4)	4.4 (3.4–5.3)	15.4	−2.2	12.8
	More than 100,000	4.1 (3.5–4.8)	5.1 (4.4–5.7)	4.8 (4.2–5.3)	24.4	−5.9	17.1

In the multivariable analysis for potential factors associated with the female homicide rates in the last triennium (2016–2018), the final model indicated positive associations between the female homicide rate and male homicides (0.05; p-value <0.001), homicides of women with a low level of education (0.02; p-value <0.001), homicides of black women (0.01; p-value <0.001), and GDP *per capita* (0.03; p-value <0.001). On the other hand, a negative association was observed for female homicides and the municipalities with more than 50,000 inhabitants (−2.26; p-value <0.001) (Table 3).

**Table 3:** Multiple linear modelling for the female homicide rate in the triennium of 2016–2018, according to explicative variables.

Models	Variables	Coefficients	Beta-standardized coefficients	p-value
Model 1	Intercept	2.298	–	0.000
	Male homicide rate	0.051	0.267	0.000
	Percentage of female homicides involving women who had a lower education level (eight years of education or less)	0.018	0.106	0.000
	Percentage of female homicides in which the victims are black	0.013	0.094	0.000
	Population greater than 50,000	−2.108	−0.109	0.000
	OVW–Office of Violence Against Women	−0.030	−0.019	0.183
	Municipal council on Women's rights	−0.268	−0.018	0.213
	Specialized services to combat violence against women	−0.231	−0.015	0.343
	Women's Police Station	0.042	0.018	0.291
	GDP *per capita*	0.029	0.098	0.000
Final model	Intercept	2.242	–	0.000
	Male homicide rate	0.050	0.262	0.000
	Percentage of female homicides involving women who had lower education level (eight years of education or less)	0.017	0.103	0.000
	Percentage of female homicides in which the victims are black	0.013	0.092	0.000
	Population greater than 50,000	−2.264	−0.117	0.000
	GDP *per capita*	0.029	0.098	0.000

Dependent variable: Average female homicide rate.

## Discussion

This study showed an overall reduction in the rate of female homicide in Brazil between 2000 and 2018, influenced mainly by the decline in deaths by firearms. However, the granularity of our assessment allowed us to map the uneven distribution of female homicides throughout the country potentially obscuring region-specific trends. The Northeast macro-region presented an increase in the homicide rate, while the Southeast showed a decrease, especially in the larger municipalities. Among the potentially associated factors, it was observed that male homicide, the high percentage of violent deaths among black women, and those with low levels of education, in addition to the low GDP *per capita*, were positively associated with female homicide, whereas larger cities were negatively associated.

The female homicide rate in Brazil remained high during the entire studied period, varying between 6.2 and 5.4 per 1000,000 women between T1 and T3. Comparatively, the global rate of female homicides in 2017 was 1.8, and in Southern Latin America, of 1.6. Likewise, the rate of female homicide in Chile in 2017 was 1.0, and in Peru, 1.2.^15^ Over the past decade, Brazil has consistently exhibited the fourth highest rate of female homicide in South America, surpassed only by Guyana, Venezuela, and Colombia.^15^ It is noteworthy that all three countries share borders with regions in Brazil that have high homicide rates, such as the state of Roraima and the northernmost part of the Amazon. This may suggest that sociocultural characteristics shared by these areas, such as drug trafficking and illegal mining, are associated with higher rates of female homicides.

Women might be killed in various settings, and not every female homicide constitutes gender-based violence. However, it is well established in the literature that most female homicides are gender motivated, which makes them related to “*the ideology of men's entitlement and privilege over women, social norms regarding masculinity, and the need to assert male control or power, enforce gender roles, or prevent, discourage or punish what is considered to be unacceptable female behaviour*”.^3^ In this sense, studies that aim to analyse data on violence against women benefit from a gender perspective, which influences both urban violence and domestic violence, with particularly profound implications for the last one.

Data from 2022 show that of 89,000 female homicides in the world, approximately 48,800 (54%) were committed by an intimate partner or a family member.^5^ This accounts for an average of one woman's violent death every 11 min within the private sphere. In Brazil, in 2022, there were 4034 female homicides, of which 1437 resulted from domestic violence, representing 35.6% of the total number of female homicides in the country during that year.^4^ Moreover, 73% of these domestic femicides were committed by an intimate partner.^4^

Analysing the mortality by region, the Brazilian female homicide rate decreased in the studied period influenced mostly by the decrease observed in the Southeast, especially in the municipalities with a population of more than 100,000 inhabitants, which had already been shown in a previous study.^16^ Additionally, Southeast was the only macro-region to show a significant relative reduction in female homicide by firearms rate in the country. One possible explanation would be that this is reflective of an overall reduction in firearm homicides in the region. An analysis from Institute for Applied Economic Research (IPEA, acronym in Portuguese) of the violence map over 10 years supports this, showing that the Southeast was the only Brazilian macro-region where all states experienced a decrease in the proportion of firearms homicides relative to total homicides, for both sexes, over the observation period.^17^ In line with the positive changes, the Southeast is the macro-region with the highest effectiveness in the implementation and management of public policies in Brazil.^18^

On the other hand, the female homicide rate increased in the Northeast macro-region, and the maps also illustrate that the border regions of the Amazon and Pantanal regions show a “high–high” standard throughout the entire study period. These regions are depicted on maps that reference drug trafficking, agricultural frontiers, land disputes, and illegal mining, among other events that turn these locations into a so-called “second state” or “lawless state”, subsequently showing higher rates of violence and homicide, which also affects the women and children who live there.^19^^,^^20^

This study also found an association between female and male homicides, as shown in previous studies,^19^^,^^21^ and suggests that structural violence, which mostly affects men, also victimises women. For example, women have become victims of narcotraffic-related homicides, be it because they are drug users or because they were murdered in revenge crimes.^22^ In this sense, territories with drug trafficking disputes, armed conflicts, and other forms of social disorder place women in a position of even greater vulnerability.^21^

During the study period, a decrease of approximately 20% was observed in homicides by firearm in Brazil. It is important to note that in 2003, the so-called *Statute of Disarmament* was approved,^23^ which hindered the civil population's access to firearms. The Statute approval can be regarded as a turning point. Prior to its implementation, the homicide rate in Brazil exhibited an average annual growth of 5.44%, however, following its enactment, this average decreased to 0.85%, representing a six-fold reduction.^24^ Notably, the Southeast, which has the best evaluation of public policy effectiveness, also showed the greatest decrease in the firearm homicide rate.^17^^,^^18^ However, in 2019, this decree suffered a serious setback. The implementation of a revised version introduced several breaches to allow civilians' ownership and carrying of firearms. The existence of a firearm in a home environment increases the risk of death of a woman who lives in a scenario of domestic violence. In 2020, for example, a nearly 5% increase was observed in the rate of female homicide by firearm in Brazil.^25^ In 2022, 69% of the female homicides and 26% of the femicides in the country were perpetrated by firearms.^4^ In this sense, the permissive armament policy adopted by the Federal Government of Brazil from 2018 to 2022 puts women at a higher risk of homicide. Our analysis spans only until 2018 and, therefore, expanding this assessment to cover the following triennium could generate important evidence to support the return of a strict, prohibitive policy for firearm ownership and carrying in Brazil.

The confirmation of the positive association among female homicides, the proportion of deaths among black women, and those with a lower level of education, in addition to the GDP *per capita*, corroborates with prior studies.^20^^,^^26^ In Brazil, Black women have twice the chance of dying by homicide than white women.^19^ The Atlas-Violence-2021 showed that, while the number of homicides among white women seems to be decreasing in recent years, the number of homicides among black women has been increasing.^27^ Reflective of its recent history of slavery, structural racism shapes the socioeconomic development in Brazil, being evident in the intersection of race and education levels as markers of socioeconomic disadvantage.^28^ In this sense, Black women and those with low levels of education are more vulnerable, representing the majority of the disadvantaged population of the country.^29^

Poverty alone does not fully explain the increased exposure to violence, but economic inequalities and social inequities render the populations more vulnerable. In addition to low income, poorer women confront deprivations, with little or no dignified opportunities for work or socialisation, which can make them more susceptible to organised crime and violent deaths.^20^ Moreover, the inequality of access to protection networks and the lack of social support can make it difficult to break the cycle of violence in which poorer women might live, increasing the risk of an escalation of both violence and femicide.^30^ In this light, gender, race, and social class can be considered social determinants of health, impacting the unequal distribution of violent acts within Brazilian society.^31^ It is therefore imperative that public policies in Brazil assume a perspective of intersectionality and be constructed in such a way as to protect the groups that are most vulnerable to fatal violence.

In this study, municipalities with less than 50,000 inhabitants presented the highest female homicide rates. Smaller towns can present a more conservative culture in such a way that women are subjected to more strict gender rules, and for this reason, they are at a higher risk of femicide as a form of punishment.^19^ In addition, access to social protection may be hindered due to the non-existence of referral services in the cascades of health care, social welfare, and public security in these municipalities.^32^

The policies geared towards protecting women have witnessed some progress in the last two decades. It is important to highlight the creation of the Secretariat of Policies for Women and the ratification of Laws 11.340/2006 (known as the Maria da Penha Law, this law specifically addresses domestic violence, establishing the definition of violence to be adopted in the legal terms and creating a legal framework for the implementation of a protective network for women) and 13.104/2015 (or Femicide Law, which establishes femicide as a qualified homicide and discusses the punishment of the aggressors). One study from IPEA^33^ identified that the Maria da Penha Law, for example, has had significant effects in the reduction of gender-related female homicides, corroborating the results from the present study concerning the decline in female homicide over the past 18 years.

However, since 2015, the continuous dismantling of women's protective mechanisms, coupled with the implementation of austerity measures such as freezing social spending, have imposed new challenges to the protection and advancement of women's rights.^34^^,^^35^ Hence, further studies are needed for an in-depth understanding of the negative repercussions of these setbacks in living conditions and health of women over the medium and long term.

This study must be discussed in light of its limitations. This was an ecological study, and, therefore, some associations presented at the population level might not have a similar representation at the individual level. Likewise, this is an observational study and inferences about causal relationships cannot be made. The GBD methodology was applied to correct the underreporting of homicide in the Brazilian Mortality System. However, pressures to avoid legal implications of homicides may lead to a misclassification of them as “unintentional injury” deaths. This can impact the *garbage* code redistribution process of undetermined intention deaths, especially in states where the SIM presents a lower quality of registration of causes of death. Another limitation concerns the quality of data in the SIM, which historically may exhibit considerable incompleteness, especially regarding the education variable.^36^ Additionally, the lack of data from the most recent years limited the analysis of the impacts that the political changes in the last period caused.

In conclusion, this study demonstrated a decrease in the female homicide rate in Brazil between 2000 and 2018, which may well represent the effectiveness of the public policies adopted by the country, mainly between 2003 and 2015. Nevertheless, this decrease did present both regional and sociodemographic discrepancies, illustrating that the vulnerability of women occurs unequally in Brazil. The violent death of women is mostly caused by domestic conflicts, but it is also influenced by changes in the urban and social contexts, such as the availability of firearms and the dynamic of drug trafficking. Thus, the gender-based violence approach needs to be intersectoral and structural, ensuring equal and fair access to rights for all women, with special attention to those whose vulnerabilities increase the risk of experiencing violence. Only through these efforts will it be possible to achieve SGD 5 of the UN 2030 Agenda, which establishes the elimination of all types of violence against all women.

## Contributors

NMV participated in conceptualization, literature search, study design, methodology, data analysis and interpretation, writing the original draft, revision, and editing of the final text.

JBS participated in conceptualization, literature search, study design, methodology, data analysis and interpretation, production of results, writing the original draft, and revision of the final text.

AMSF contributed to conceptualization, literature search, methodology, production of results, formal analysis, and writing and revising the final text.

PHC participated in the literature search, formal analysis, writing, and revising the final text.

SR participated in the data analysis and interpretation, funding acquisition and project administration, and writing and revising the final text.

CS contributed to the data interpretation, writing, revising, and editing of the final text.

CSG participated in review and editing of the final text.

LSF participated in data interpretation, writing, revising, and editing the final text.

EG participated in writing, revising, and editing the final text.

ALPR participated in the validation, review, and editing of the final text.

DCM contributed to conceptualization, data curation, funding acquisition, methodology, supervision, review, and editing of the final text.

JBS, AMSF, ALPR and DCM accessed and verified the underlying data. All authors participated in the decision to submit it.

## Data sharing statement

This study used secondary data from public databases. All the databases are in the public domain and can be accessed on the internet at the following websites:

Brazilian Mortality Information System: https://svs.aids.gov.br/daent/centrais-de-conteudos/dados-abertos/sim/.

Municipalities GDP *per capita*: https://www.ibge.gov.br/estatisticas/economicas/contas-nacionais/9088-produto-interno-bruto-dos-municipios.html.

Municipalities population estimate: https://sidra.ibge.gov.br/pesquisa/estimapop/tabelas.

Basic Municipal Information Survey (MUNIC, in Portuguese), 2018: https://www.ibge.gov.br/estatisticas/sociais/saude/10586-pesquisa-de-informacoes-basicas-municipais.html?edicao=18195&t=downloads.

Brazilian female population estimate, by municipality: http://tabnet.datasus.gov.br/cgi/deftohtm.exe?ibge/cnv/popsvsbr.def.

Standard population from GBD: https://ghdx.healthdata.org/gbd-2019.

## Editor's note

The Lancet Group takes a neutral position with respect to territorial claims in published maps and institutional affiliations.

## Declaration of interests

The authors have no conflicts of interest in this work.

## Appendix A Supplementary data

Translated summary
