# Supplementary material for: Female homicides in Brazil: global burden of disease study, 2000–2018
Source: Lancet Reg Health Am. 2024 Nov 7;40:100935. doi: 10.1016/j.lana.2024.100935 (PMC11570984; doi:10.1016/j.lana.2024.100935)
Supplement: Translated summary [file mmc1.docx]

***Editorial Disclaimer:*** *This translation in Portuguese was submitted by the authors and we reproduce it as supplied. It has not been peer-reviewed. Our editorial processes have only been applied to the original abstract in English, which should serve as a reference for this manuscript.*

**Resumo**

**Contexto:** Homicídios femininos são um relevante problema de Saúde Pública e sua distribuição espacial pode evidenciar vulnerabilidades socioeconômicas. O presente estudo objetivou analisar as tendências temporal e espacial dos homicídios femininos no território brasileiro e investigar os possíveis fatores socioeconômicos e demográficos associados a esse agravo.

**Metodos:** Estudo ecológico, descritivo e analítico que investigou a taxa de homicídios femininos padronizada por idade em todos os municípios brasileiros entre 2000 e 2018, dividida em três períodos. Análises espaciais e temporais foram realizadas utilizando o Índice Global de Moran e LISA para identificar agrupamentos de altas e baixas taxas. As taxas também foram calculadas por tamanho populacional e meios de violência nas macrorregiões. No último período, um modelo de regressão linear multivariada analisou a associação das taxas de homicídios femininos com fatores sociais, econômicos e geográficos.

**Resultados:**  A taxa de homicídios feminino no Brasil manteve-se alta ao longo de todo o período estudado, com tendências diferentes entre as regiões. Entre os potenciais fatores associados, foi observado que homicídios masculinos, alta porcentagem de mortes violentas entre mulheres negras ou com baixa escolaridade, além de baixo Produto Interno Bruto *per capita*, foram positivamente associados ao homicídio feminino, enquanto cidades maiores tiveram associação negativa.

**Interpretação:** Esses achados mostram que o Brasil é um país com alto risco de homicídios para mulheres. No entanto, a vulnerabilidade das mulheres ocorre de forma desigual no país. As mortes violentas de mulheres são causadas principalmente por conflitos domésticos, mas também são influenciadas por mudanças nos contextos urbano e social.

**Financiamento:** Esse projeto foi financiado pela Bill & Melinda Gates Foundation.

**Palavras-chave:** Violência contra as mulheres; Violência de gênero; Brasil; Homicídio; Vigilância em Saúde Pública
